# Identification of the Dominant Rainfall Index and Evolution of Multi-Factor Driving Mechanisms for Landslide Activity in Hong Kong (1990–2024)

**DOI:** 10.3390/s26051430

**Published:** 2026-02-25

**Authors:** Jiaqi Wu, Zelang Miao, Yaopeng Xiong, Zefa Yang, Xiangqian Shen

**Affiliations:** 1 Laboratory of Geo-Hazards Perception, Cognition and Predication, School of Geoscience and Info-Physics, Central South University, Changsha 410083, China235007014@csu.edu.cn (Y.X.);; 2 School of Computer Science, Wuhan University, Wuhan 430072, China; shenxq@whu.edu.cn; 3 Kunming Prospecting Design Institute of China Nonferrous Metals Industry Co., Ltd., Kunming 650051, China

**Keywords:** landslide, grey relational analysis, rainfall index, optimal-parameter geographical detector, spatiotemporal evolution

## Abstract

Revealing the spatiotemporal driving mechanisms of landslide activity is fundamental to improving long-term landslide hazard management and risk mitigation in mountainous cities. Focusing on landslide events in Hong Kong from 1990 to 2024, this study develops an integrated framework at the slope-unit scale that combines rainfall index optimization with multi-factor spatiotemporal driving analysis. First, Grey Relational Analysis (GRA) is employed to systematically evaluate the spatiotemporal associations between landslide occurrences and six commonly used rainfall indices, aiming to obtain a consistent and robust representation of rainfall triggering conditions. Subsequently, the Optimal-Parameter Geographical Detector (OPGD) model is introduced to quantitatively assess the explanatory power of individual factors—covering geological, topographic, hydro-meteorological, and human-related variables—as well as their pairwise interactions, thereby revealing the spatiotemporal evolution of landslide driving factors and their multi-factor coupling mechanisms over a 35-year period. The results indicate that the maximum 3-day cumulative rainfall index (RX3day) consistently exhibits the strongest association across different resolution parameter settings and is identified as the dominant rainfall indicator representing dynamic landslide triggering. Geological conditions and topographic factors constitute a stable background controlling the spatial heterogeneity of landslides throughout the entire study period, whereas the explanatory power of RX3day increases markedly after around 2000, gradually emerging as a primary dynamic driving factor of landslide activity. Interaction detection further demonstrates that landslide occurrence is mainly governed by nonlinear enhancement effects among multiple factors, with “geology–topography” and “rainfall–topography/geology” interactions showing the highest explanatory power, and rainfall-related interactions exhibiting continuous strengthening over time. Overall, the spatiotemporal distribution of landslides in Hong Kong is jointly controlled by long-term stable geological–topographic conditions and increasingly intensified extreme rainfall forcing.

## 1. Introduction

Landslides are among the most widespread and destructive geological hazards, occurring particularly frequently in mountainous and coastal regions characterized by pronounced topographic relief and concentrated rainfall [1]. Such hazards not only pose severe threats to human life and property but also exert profound impacts on infrastructure operation and regional socioeconomic development [2]. With the background of global climate change, the frequency and intensity of extreme rainfall events have increased markedly. This trend is especially evident in Southeast Asia and other humid, monsoon-dominated coastal mountainous regions, where it has been shown to be closely associated with rising landslide occurrence rates [3]. The combined effects of rainfall, topographic conditions, geological and geotechnical characteristics, and human disturbances substantially enhance landslide susceptibility and complicate triggering processes, making landslides one of the geological hazards that require sustained and focused attention [4].

Hong Kong is located in a typical subtropical coastal mountainous environment, characterized by steep terrain, highly fractured geological structures, and frequent intense rainfall, making it one of the most landslide-prone urban regions worldwide. In recent years, extensive research has been conducted by both domestic and international scholars on landslide susceptibility assessment, hazard mapping, and predictive modeling in Hong Kong. Based on historical landslide inventories and multi-source geospatial data (e.g., topography, geology, hydrology, and rainfall), a wide range of statistical methods, machine learning, and deep learning approaches have been applied, substantially improving the accuracy of landslide spatial prediction and providing critical support for hazard zoning and early warning systems. For example, Wang et al. [5] proposed an artificial intelligence–based object-oriented landslide susceptibility assessment method and produced high-resolution landslide susceptibility maps for Hong Kong. Chen et al. [6] developed a feature-fusion CPCNN-ML model for landslide susceptibility mapping in Lantau Island, Hong Kong, demonstrating that multi-source feature integration can significantly enhance deep learning performance. Lyu et al. [7] incorporated mitigation strategies into machine learning models by generating non-landslide samples in stabilized areas and comparing scenarios with and without mitigation measures, showing that mitigation works can effectively reduce landslide susceptibility in Hong Kong. Beyond landslide susceptibility mapping, increasing attention has been paid to landslide initiation mechanisms and their driving factors. Existing studies indicate that landslide activity in Hong Kong is jointly controlled by relatively stable background conditions (such as geology and topography) and highly dynamic triggering factors, among which rainfall is widely recognized as the most critical external trigger [8,9]. To characterize rainfall processes, various quantitative rainfall indices based on cumulative amounts over different durations and percentile thresholds have been proposed. For instance, Ko and Lo [10] introduced a normalized maximum 24 h rolling rainfall index, which normalizes the maximum 24 h rainfall during storm events by the local mean annual rainfall, and applied it to rainfall-induced landslide susceptibility analysis for natural terrain units in Hong Kong. Li et al. [11] proposed the normalized maximum rolling rainfall (NMRR) index, normalizing the maximum 4 h and 24 h rolling rainfall by multi-year mean annual rainfall, and incorporated it as a key input feature in machine learning models to improve the prediction accuracy of rainfall-induced landslides in Hong Kong. Ren et al. [12] developed the maximum rolling rainfall index (MRRI) based on real-time rolling cumulative rainfall over multiple durations (2–24 h) and integrated it as a dynamic factor into a random forest model to achieve spatiotemporal landslide susceptibility mapping in Hong Kong. Ma et al. [13] introduced annual extreme rainfall days (AERD) as a key index, revealing the dominant role of extreme rainfall intensity in the dynamic landslide susceptibility of Lantau Island over the past three decades, and demonstrating that its importance is modulated by both climate change and mitigation engineering works. These quantitative rainfall indices have been widely applied in studies of rainfall–landslide relationships and predictive modeling.

Although the above studies have achieved important progress, notable limitations remain in characterizing long-term spatiotemporal driving mechanisms. First, most existing studies rely on relatively static landslide susceptibility models and single-period or short-term datasets, making it difficult to systematically reveal the spatiotemporal variability and long-term evolution of landslide driving factors. Although Qiu et al. [14] introduced spatiotemporal consistency analysis in landslide susceptibility studies for Hong Kong, the spatiotemporal variability of driving factors and their interaction mechanisms were not comprehensively examined. Second, current interpretations of landslide driving mechanisms are largely based on post hoc model outputs, such as feature importance rankings or explainable algorithms (e.g., SHAP values). These interpretations strongly depend on specific model structures, algorithmic assumptions, and variable selection, and therefore may not directly reflect the intrinsic contributions of individual driving factors [7,13,15].With respect to rainfall-related factors, although a variety of quantitative rainfall indices have been proposed, strong correlations commonly exist among different indices. When multiple indices are simultaneously introduced into analyses without discrimination, issues such as multicollinearity, information redundancy, and unstable interpretations of rainfall–landslide relationships may arise [16,17,18,19]. Although Yan et al. [20] employed grey relational analysis combined with multiple extreme rainfall indices to further address the complex relationships between rainfall indicators and landslide density, their study lacked an in-depth spatial analysis of rainfall indices and landslides. Overall, despite the availability of relatively complete long-term landslide inventory data for Hong Kong, an integrated analytical framework that can systematically compare multiple quantitative rainfall indices in both temporal and spatial dimensions and effectively link them to landslide distributions is still lacking. This gap constrains the scientific identification of the most effective triggering rainfall indicators and weakens a comprehensive understanding of regional landslide driving mechanisms under spatiotemporal dependence conditions [21,22,23,24].

In addition, landslide activity in Hong Kong has exhibited pronounced spatiotemporal heterogeneity over the past three decades, driven by the combined effects of climate change, increasing frequency of extreme rainfall events, land-use transformation, and other natural and anthropogenic factors [25]. This complex background, together with the limitations of existing studies, highlights the necessity of developing a unified analytical framework that integrates static geological and topographic conditions with dynamic climatic and environmental factors at the slope-unit scale. Such a framework enables long-term, multi-factor, and cross-stage analyses of landslide driving mechanisms, thereby facilitating a deeper understanding of the spatiotemporal evolution of landslide activity and its dominant controlling processes in Hong Kong [26].

Based on the above analysis, this study focuses on landslide events in Hong Kong from 1990 to 2024 and develops an integrated research framework at the slope-unit scale that combines optimal selection of quantitative rainfall indices with multi-factor spatiotemporal driving mechanism analysis. The objectives of this study are twofold: (1) to systematically compare the associations between multiple candidate quantitative rainfall indices and landslide activity from a spatiotemporal perspective, and to identify the dominant rainfall index with the strongest triggering effectiveness for landslides, thereby addressing uncertainties arising from inconsistent rainfall representations and multicollinearity in long-term analyses; and (2) to quantitatively identify the dominant driving factors of landslide activity over the past 35 years and to characterize their spatiotemporal evolution and interaction mechanisms across different temporal stages. Methodologically, the framework first applies grey relational analysis at the slope-unit scale to objectively compare and select candidate rainfall indices from a spatiotemporal perspective, thereby identifying the dominant rainfall index most strongly associated with landslide occurrence. Subsequently, the optimal parameter-based geographical detector is introduced to jointly analyze the selected dominant rainfall index together with other geological, topographic, hydro-meteorological, and human activity factors, enabling quantitative assessment of the single-factor explanatory power and pairwise interaction effects of these factors, as well as their spatiotemporal evolution over the entire 35-year study period (divided into seven consecutive five-year stages). This study provides a scientific basis for landslide hazard prevention, urban planning, and risk management in Hong Kong.

## 2. Study Area and Data Sources

### 2.1. Study Area and Landslide Inventory

**Figure 1:**
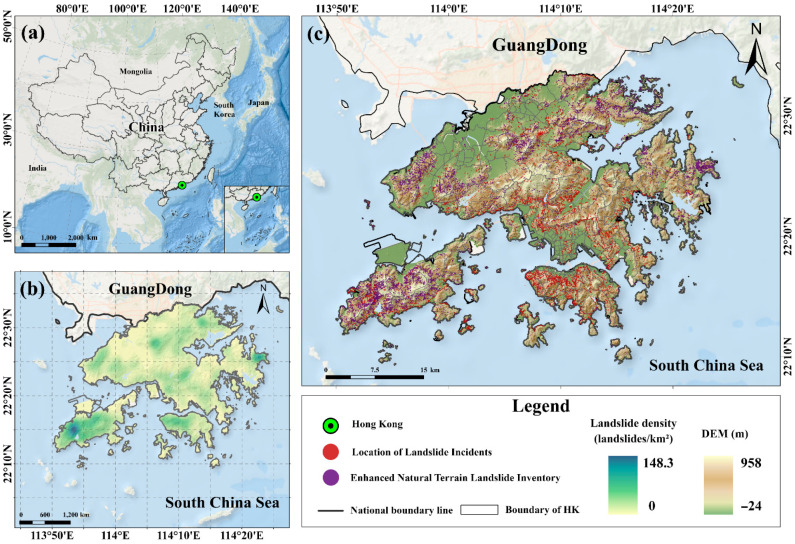
Map of the study area. (**a**) Map of China; (**b**) Map of landslide density in Hong Kong; (**c**) DEM and the landslide inventory of the study area.

Hong Kong is located in the subtropical hilly coastal region of southeastern China (Figure 1). The territory is characterized by dense mountainous terrain, steep slopes, and complex geological structures, with widespread distributions of granite, volcanic rocks, and their weathered products, collectively forming geological and geomorphological conditions favorable for landslide development [27]. The region is strongly influenced by the East Asian monsoon, and intense, short-duration rainstorms frequently occur during summer, serving as the primary meteorological trigger for shallow landslides [28]. In addition, as one of the most densely populated cities in the world, Hong Kong has long been constrained by limited developable land, forcing extensive residential areas, road networks, and public infrastructure to be constructed on or adjacent to hillslopes, which substantially increases landslide risk. Landslides in Hong Kong are characterized by high occurrence frequency, large variability in scale, and strong destructive potential, and their sudden onset often poses serious threats to human life, property, and the operation of critical infrastructure [29]. Spatially, landslides are mainly concentrated in the natural mountainous areas of the New Territories, including the Islands District (particularly Lantau Island), Tai Po, Tsuen Wan, Tuen Mun, and Sai Kung, as well as in residential areas and public facilities located adjacent to hillslopes.

Figure 1b,c illustrate the study area of Hong Kong and two authoritative landslide datasets used to characterize landslide density and spatial distribution patterns from 1990 to 2024. The first dataset is the Enhanced Natural Terrain Landslide Inventory (ENTLI), compiled by the Geotechnical Engineering Office (GEO) under the Civil Engineering and Development Department (CEDD) of the Hong Kong Government. This dataset was primarily established through systematic interpretation of aerial photographs and includes a large number of both recent and relict natural terrain landslides, thereby reflecting the spatial distribution and geomorphological characteristics of landslides in Hong Kong’s natural terrain [30]. The second dataset is the Location of Landslide Incidents, which is continuously updated by CEDD/GEO and publicly available through the Common Spatial Data Infrastructure (CSDI) and DATA.GOV.HK platforms. This dataset records landslide incidents including both natural terrain landslides and man-made slope failures, and provides detailed attribute information such as spatial location, failure scale, and consequences [31]. Two landslide inventories were harmonized in terms of coordinate systems and attribute structures, and duplicate landslide points were identified and removed through spatial proximity analysis. Ultimately, a spatially consistent and temporally continuous integrated landslide inventory for the period 1990–2024 was constructed, providing a unified data foundation for subsequent rainfall–landslide association analyses and multi-factor driving mechanism investigations at the slope-unit scale.

### 2.2. Landslide Conditioning Factors

**Figure 2:**
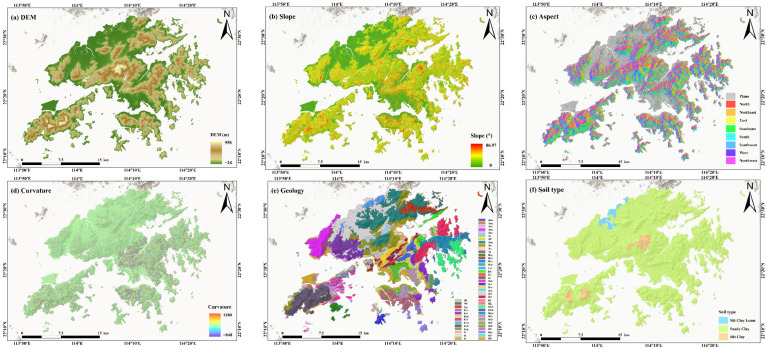

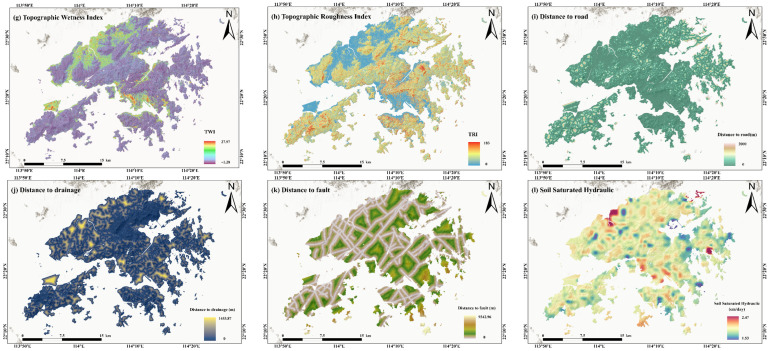
Static landslide conditioning factors: (**a**) DEM; (**b**) slope; (**c**) aspect; (**d**) curvature; (**e**) geology; (**f**) soil type; (**g**) TWI; (**h**) TRI; (**i**) distance to road; (**j**) distance to drainage; (**k**) distance to fault; (**l**) soil saturated hydraulic.

**Figure 3:**
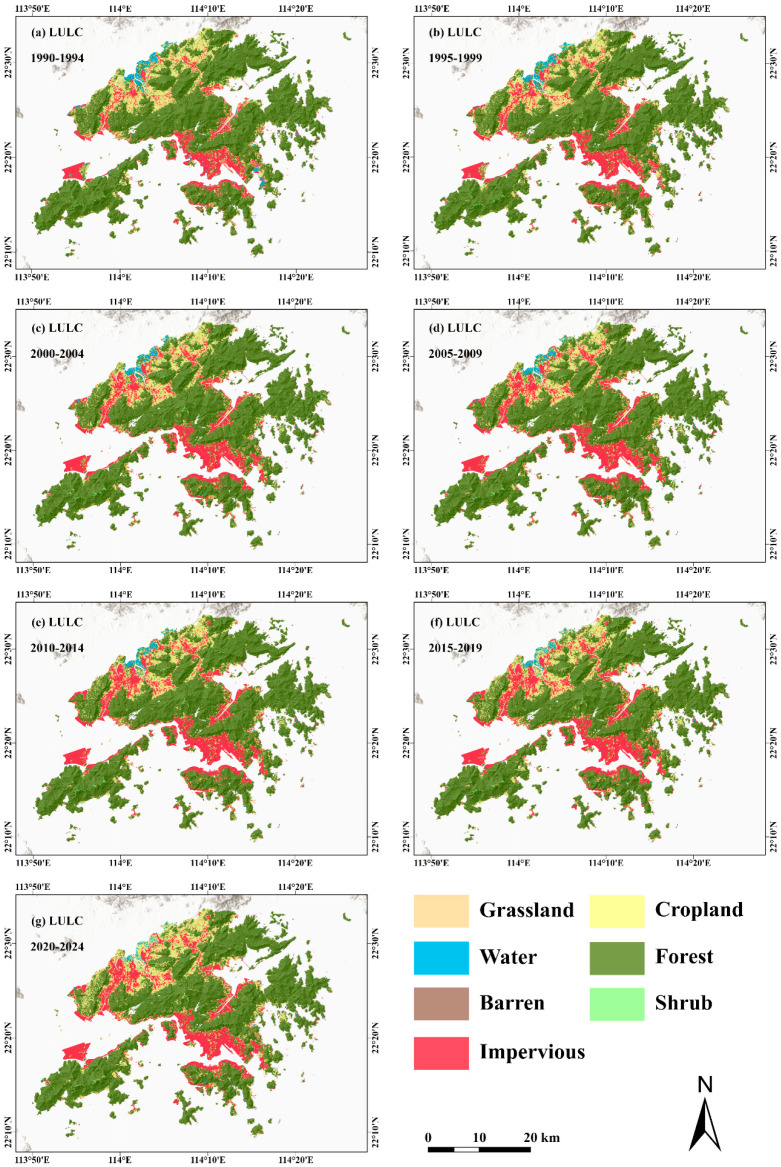
(**a**–**g**) Land use/land cover (LULC) maps at five-year intervals during 1990–2024.

This study selected four categories of geospatial data related to landslide hazards in the study area, including topographic and geomorphological characteristics, geological conditions, hydro-meteorological factors, and human activities. Specifically, the selected variables include static factors such as DEM, slope, aspect, curvature, lithology, soil type, topographic wetness index (TWI), terrain roughness index (TRI), distance to roads, distance to rivers, distance to faults, and saturated hydraulic conductivity, as well as dynamic factors including land use/land cover (LULC) and rainfall. These data were mainly derived from topographic surveying, remote sensing observations, and meteorological observation products. Detailed information on the datasets is provided in Table 1, and the spatial distributions of all original geospatial datasets are shown in Figure 2. Specifically, the DEM was obtained from the B50K topographic map dataset acquired by the Lands Department of Hong Kong based on airborne laser scanning, which provides elevation data derived from topographic surveying and remote sensing and serves as the basis for the calculation of terrain-related factors. Slope, aspect, curvature, topographic wetness index, and terrain roughness index were all derived from the DEM. Distances to roads, rivers, and faults were generated based on vector data of roads, drainage systems, and faults in Hong Kong using the Euclidean distance method, with the DEM used as the spatial reference to produce corresponding raster layers. Among these time-varying landslide assessment factors, rainfall data were obtained from the CHIRPS dataset, which was generated by integrating observations from multiple satellite-based infrared and microwave sensors with global ground-based rain gauge data. Based on the dominant rainfall index identified through gray relational analysis (GRA) as having the highest association, the mean value of the dominant rainfall index over a five-year period was calculated to characterize regional-scale rainfall conditions. The land use/land cover (LULC) data were obtained from the CLCD dataset, which was developed based on multispectral remote sensing observations from the Landsat satellite series. In this study, the LULC data were slightly adjusted according to actual conditions, and the category with the highest frequency over the five-year period was selected as the representative value (Figure 3).

Landslide conditioning factors were resampled to raster data with a spatial resolution of 5 m and projected to the Hong Kong 1980 Grid coordinate system, which is most suitable for the geographical location of Hong Kong. In subsequent data processing, slope units were used as the analysis scale. For continuous geospatial data, mean values within each slope unit were calculated to represent the overall environmental characteristics of the slope unit, whereas for discrete geospatial data, the category with the highest frequency within each slope unit was used as the representative value.

## 3. Methodology

**Figure 4:**
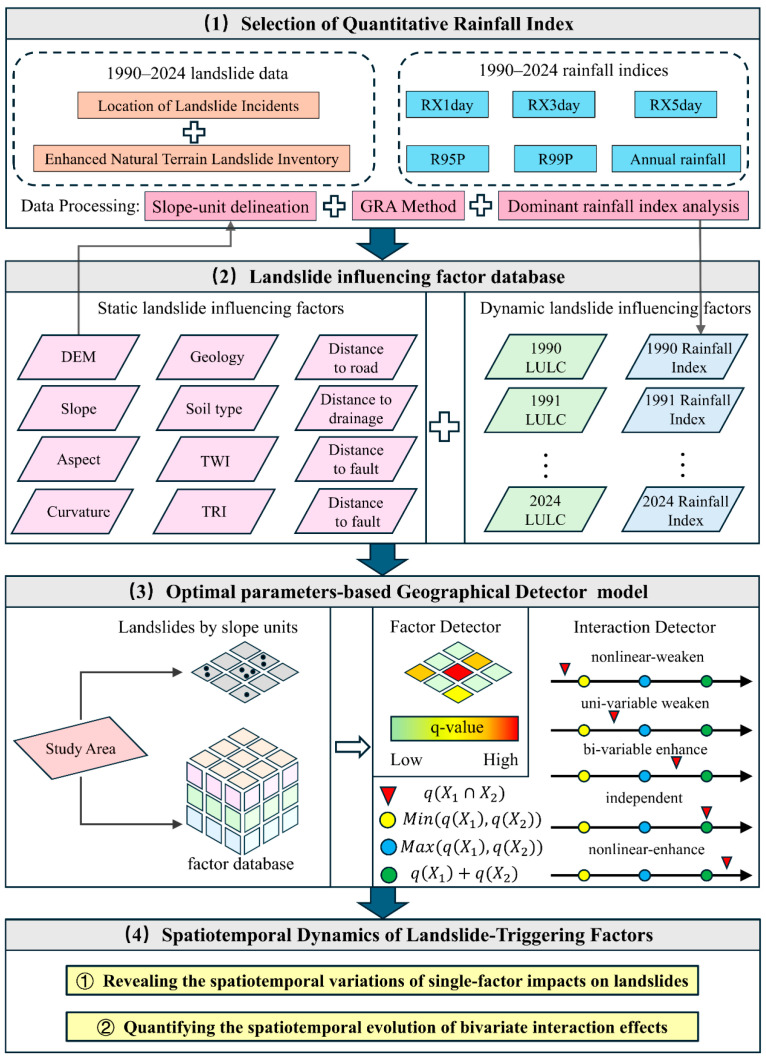
Overall workflow flowchart of the study.

This study focuses on landslide events in Hong Kong from 1990 to 2024. Data preprocessing was conducted in ArcGIS Pro, while the GRA and GeoDetector modeling and analyses were performed in Python (version 3.12.3). Given that slope units are widely adopted in landslide research and can effectively represent geomorphic structures and hillslope hydrological processes, a comprehensive analytical framework integrating dominant rainfall index selection and multi-factor spatiotemporal driving mechanism analysis was developed at the slope-unit scale. Slope units in the study area were delineated using the r.slopeunits module [32], which is based on automated extraction of terrain watersheds and flow lines; this module generates fundamental terrain attributes such as flow direction and flow accumulation from DEM data to identify geomorphologically homogeneous slope units at the catchment scale. The analysis first applied Grey Relational Analysis (GRA) to systematically compare the spatiotemporal associations between multiple candidate rainfall indices and landslide activity, thereby identifying the dominant rainfall index most strongly associated with landslide occurrence and reducing the effects of inconsistent rainfall representation and indicator collinearity in long-term analyses. On this basis, the Optimal Parameter-Based Geographical Detector (OPGD) was introduced to conduct long-term temporal driving analysis of static and dynamic factors, including geological, topographic, hydro-meteorological, and human-activity variables, quantitatively assessing the explanatory power of individual factors and their pairwise interaction enhancement effects. Through this integrated framework, the spatiotemporal evolution characteristics of landslide activity in Hong Kong over the past 35 years and its dominant driving mechanisms were systematically characterized. The overall research workflow is illustrated in Figure 4.

### 3.1. Identification of the Dominant Rainfall Index

Based on the constructed integrated landslide inventory dataset, this study combined landslide events with the CHIRPS (Climate Hazards Group InfraRed Precipitation with Station data) rainfall product provided by the Climate Hazards Group to quantify the relationship between rainfall conditions and landslide occurrence [33]. Using slope units as the analysis scale, spatial averaging was applied to the CHIRPS rainfall data, and several commonly used rainfall indices were calculated, including Rx1day, Rx3day, Rx5day, R95p, R99p, and annual rainfall. These rainfall indices have been widely applied in studies of landslide susceptibility and triggering mechanisms. Considering the potential strong correlations among different rainfall indices, and to avoid the influence of multicollinearity on the analytical results while systematically characterizing the response relationships between different rainfall characteristics and landslide activity, GRA was employed to quantitatively evaluate the spatiotemporal associations between each rainfall index and landslide density during the period 1990–2024 [20,34]. Compared with traditional correlation analysis methods, GRA does not rely on assumptions regarding variable distributions nor require linear relationships between variables, making it suitable for association analysis under multi-indicator and nonlinear conditions. By comparing the grey relational degrees between different rainfall indices and landslide activity sequences, the rainfall index playing a dominant role in landslide occurrence was identified for subsequent driving mechanism analysis. The calculation formula of GRA is as follows (Equation (1)):

(1) $$\gamma (y_{0j},x_{ij})=\frac{\begin{array}{l} \min\left\{|y_{0j}-x_{ij}|,\;i=1,2,\ldots ,m;\;j=1,2,\ldots ,n\right\}\\ +\tau \max\left\{|y_{0j}-x_{ij}|,\;i=1,2,\ldots ,m;\;j=1,2,\ldots ,n\right\} \end{array}}{\Delta_{ij}+\tau \max\left\{|y_{0j}-x_{ij}|,\;i=1,2,\ldots ,m;\;j=1,2,\ldots ,n\right\}}$$

where $\gamma (y_{0j},x_{ij})$ denotes the grey relational coefficient between $x_{ij}$ and $y_{0j}$. A larger value of the grey relational coefficient indicates a higher degree of similarity or closeness between $x_{ij}$ and $y_{0j}$. τ represents the distinguishing coefficient.

### 3.2. Optimal Parameter-Based Geographical Detector

To systematically analyze the differentiated driving mechanisms of landslide spatiotemporal patterns in Hong Kong during 1990–2024, and to reduce the sensitivity of traditional geographical detectors to discretization parameter settings for continuous variables, this study introduced the Optimal Parameter-based Geographical Detector at the slope-unit scale to automatically optimize the spatial discretization of raster-based landslide influencing factors [35]. The geographical detector is a statistical method designed to identify spatial heterogeneity in geographical phenomena and to quantify the contributions of their driving factors, and it has been widely applied to characterize the relationships between environmental factors and landslide spatial distributions. Owing to its advantages in spatial differentiation analysis, this method enables the quantitative evaluation of the explanatory power of individual influencing factors on landslide occurrence, thereby facilitating the identification of dominant driving mechanisms of landslide susceptibility. The geographical detector measures the degree to which an influencing factor explains the spatial heterogeneity of the target variable using the q statistic, and the calculation formula of the geographical detector is expressed as follows (Equation (2)):

(2) $$q=1-\frac{1}{N\sigma^{2}}\sum_{m=1}^{L}N_{m}\sigma_{m}^{2}$$

where q represents the explanatory power of a regional geographical environmental factor; m = 1, 2, …, L denotes the number of categories; $N_{m}$ and N represent the number of slope units in category m and the total number of slope units in the entire study area, respectively; and $\sigma^{2}$ denotes the variance of the indicator. The value of q ranges from 0 to 1, with larger q values indicating a stronger ability of the factor to explain the spatial heterogeneity of landslides. In this study, the q statistic is used to quantify the explanatory power of different influencing factors on the spatial differentiation of landslides in Hong Kong during 1990–2024, as well as their temporal evolution, thereby characterizing the relative dominance of driving factors across different time stages and their dynamic changes.

Based on the single-factor detection results, interaction detection was further conducted to evaluate whether the joint effects of any two influencing factors enhance or weaken their explanatory power for landslide occurrence. By comparing the q values of individual factors with those obtained after factor interaction, the interaction types between factors can be identified, including nonlinear enhancement, bi-factor enhancement, independence, or weakening effects [36]. This analysis facilitates the identification of compound driving mechanisms of landslide formation under different combinations of topographic, geological, and climatic conditions, and contributes to a deeper understanding of the origins of landslide spatiotemporal heterogeneity.

## 4. Experimental Results

### 4.1. Spatiotemporal Distribution and Evolutionary Trends of Landslides in Hong Kong from 1990 to 2024

**Figure 5:**
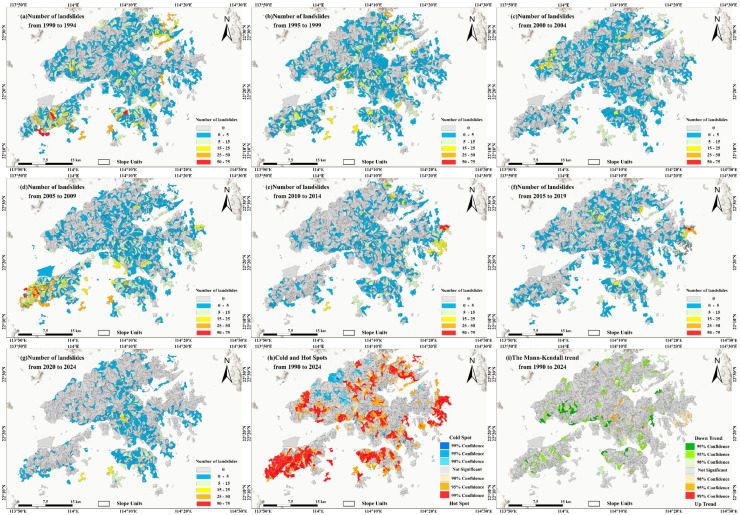
(**a**–**g**) Landslide distribution maps at the slope-unit scale (1990–2024); (**h**) Getis–Ord Gi* hotspot–cold spot map; (**i**) Mann–Kendall trend map of landslide counts.

Based on the integrated landslide inventory, spatially non-overlapping landslide records from 1990 to 2024 were extracted to construct a temporally continuous landslide sample dataset. The study area was divided into 4431 slope units, and the number of landslides occurring within each unit was counted for each five-year period at the slope-unit scale. Based on these unit-based landslide statistics, the spatial clustering characteristics of landslide activity in Hong Kong over the past 35 years and their temporal evolution were comprehensively analyzed. Figure 5a–g illustrate the spatial distribution patterns of landslides at the slope-unit scale for successive five-year intervals during 1990–2024. Figure 5h presents the results of the Getis–Ord Gi* hotspot analysis [37], showing that 981 slope units (22.1%) were identified as hotspots and 359 units (8.1%) as cold spots, while the remaining 3091 units (69.8%) did not pass the significance test. Figure 5i shows the results of the Mann–Kendall trend test of landslide counts at the slope-unit scale [38], indicating that 28 units (0.6%) exhibited a significant increasing trend, 282 units (6.4%) showed a significant decreasing trend, and the remaining 4121 units (93.0%) exhibited no significant trend. Table 2 cross-tabulates the number of slope units under different spatial patterns by integrating the hotspot and trend analysis results. The results indicate that 13 units (0.29%) were classified as “hotspot–increasing”, 158 units (3.57%) as “hotspot–decreasing”, and 810 units (18.28%) as “hotspot–stable”. In addition, 15 units (0.34%) transitioned from non-significant to an increasing trend, while cold-spot units were predominantly stable, accounting for 8.08% of all slope units.

### 4.2. Spatiotemporal Associations Between Rainfall Indices and Landslide Activity

**Figure 6:**
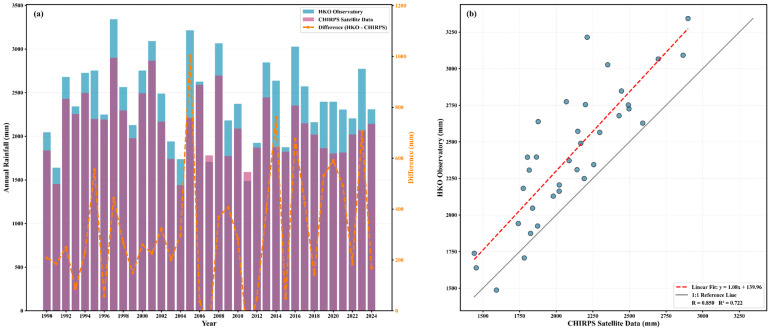
Comparison and correlation analysis of annual rainfall between HKO Observatory and CHIRPS satellite data in Hong Kong (1990–2024). (**a**) Annual rainfall comparison with difference curve; (**b**) Correlation scatter plot with linear regression.

To verify the applicability of the CHIRPS satellite rainfall data in the Hong Kong region, this study conducted a comparative analysis between rainfall observations from the Hong Kong Observatory (HKO) and CHIRPS rainfall data during the period from 1990 to 2024 (Figure 6). The results indicate that, over the 35-year study period, the two data sources exhibit highly consistent interannual variation patterns, with a correlation coefficient of 0.85, suggesting that the CHIRPS data can reliably reflect rainfall variability in the Hong Kong region. The overlaid bar chart analysis shows that CHIRPS data are generally slightly lower than the HKO observations, and this systematic bias remains relatively stable at the interannual scale. The difference curve further indicates that the interannual discrepancies between the two datasets mainly range from −100 mm to 1000 mm, with more pronounced differences observed in 2005 and 2014. These discrepancies may be associated with the spatial heterogeneity of extreme rainfall events and the representativeness limitations of ground-based observation stations. Despite the presence of systematic bias, the two datasets demonstrate good consistency in characterizing interannual rainfall variation patterns, particularly in identifying wet years (e.g., 1997, 2001, and 2006) and dry years (e.g., 1991, 2004, and 2011). These results indicate that CHIRPS v3 data exhibit good applicability in the Hong Kong region, and their continuous spatial coverage can effectively compensate for the limitations of station-based observations in terms of spatial distribution. Therefore, CHIRPS v3 data can be considered a reliable data source for subsequent long-term rainfall analysis and investigations of landslide driving mechanisms.

**Figure 7:**
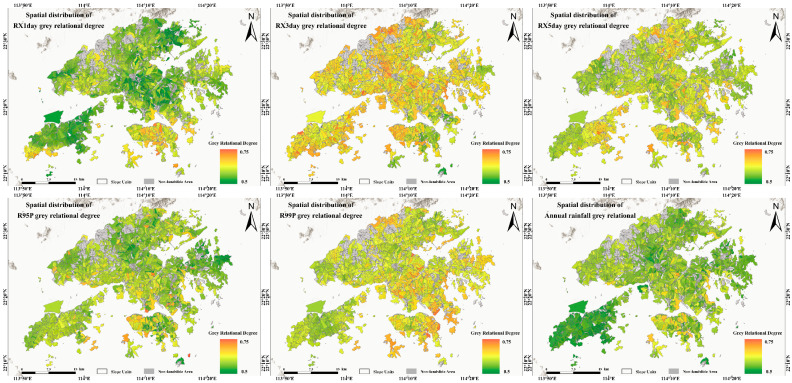
GRA relational grades between six quantified rainfall indices and landslides.

To identify the dominant rainfall index most closely associated with landslide occurrence in the study area, this study conducted Grey Relational Analysis between landslide activity and six rainfall indices at the slope-unit scale for the period 1990–2024. The results show that, among the 4431 slope units delineated in the study area, 1923 units (43.4%) experienced landslide occurrences during the study period, and these units were included in the subsequent correlation analysis. The temporal association between landslide frequency at the slope-unit scale and each rainfall index was quantified using GRA with a distinguishing coefficient of τ = 0.5, which provides a balance between sensitivity to sequence differences and result stability [39]. Figure 7 illustrates the distribution of grey relational grades for the six rainfall indices—RX1day, RX3day, RX5day, R95p, R99p, and annual rainfall—at the slope-unit scale. Overall, for slope units with recorded landslides, the grey relational grades of the rainfall indices are mainly distributed within the range of 0.50–0.75, indicating moderate to strong associations. Among them, RX3day exhibits the highest mean relational grade (0.63 ± 0.032), followed by R99p (0.62 ± 0.029), suggesting that short-duration accumulated intense rainfall and extreme precipitation events are more strongly associated with landslide occurrence. RX5day ranks third with a mean relational grade of 0.60 ± 0.027. In contrast, R95p (0.58 ± 0.031), RX1day (0.57 ± 0.035), and annual rainfall (0.57 ± 0.028) show relatively lower levels of association.

**Figure 8:**
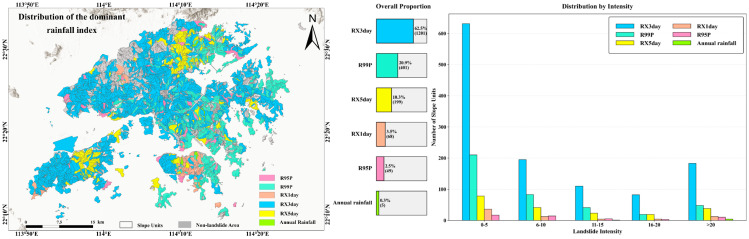
Spatial distribution and statistics of dominant rainfall index.

To reveal the dominant role of different rainfall indices in landslide triggering and their associated spatial heterogeneity, the results of the Grey Relational Analysis (GRA) were further used to identify the dominant rainfall index for each of the 1923 slope units that experienced landslides, and to examine its correspondence with landslide activity intensity (Figure 8). The results indicate that RX3day is identified as the dominant rainfall index in 1201 slope units, accounting for 62.5% of all landslide-affected units, demonstrating a clear and prevailing dominance. The second most dominant index is R99p, corresponding to 401 slope units (20.9%). Together, these two indices account for 83.4% of the total, indicating that the rainfall–landslide relationship in the study area is primarily controlled by short-duration accumulated rainfall and extreme precipitation processes. The remaining rainfall indices exhibit relatively weak dominance, with RX5day, RX1day, R95p, and annual rainfall accounting for 10.3%, 3.5%, 2.5%, and less than 1% of slope units, respectively. In terms of spatial distribution patterns (Figure 8, left), areas dominated by RX3day exhibit large-scale spatial continuity and pronounced clustering across the study area. In contrast, the dominant zones of the other five rainfall indices are mainly characterized by scattered distributions or small, patchy patterns, reflecting spatial differences in the controlling effects of distinct rainfall regimes on landslide occurrence.

At the slope-unit scale, slope units with landslide occurrences were further classified into five classes (0–5, 6–10, 11–15, 16–20, and >20 occurrences) according to the number of landslides, and the distribution characteristics of the dominant rainfall indices under different landslide activity intensity levels were analyzed (Figure 8, right). The results indicate that RX3day maintains an absolute dominant position across all landslide activity intensity classes, and its proportion remains generally stable. R99p and RX5day act as secondary dominant indices, and their proportions exhibit certain fluctuations among different classes. In the low to moderate activity intensity classes (0–10 occurrences), RX3day accounts for the highest proportion, while the combined proportion of R99p and RX5day is approximately 30%, indicating that short-duration accumulated rainfall has strong explanatory power for landslide activity at this stage. As landslide activity intensity increases (>10 occurrences), RX3day remains dominant, whereas the proportion of RX5day shows a gradual increasing trend, and the dominant proportion of R99p decreases accordingly. Overall, during the period 1990–2024, landslide activity in Hong Kong shows the strongest response to short-duration accumulated rainfall (RX3day), which occupies a dominant position across all landslide activity intensity classes. The extreme precipitation index R99p acts as an important secondary controlling factor in some regions and periods. In contrast, the dominant roles of RX5day, RX1day, R95p, and annual rainfall are relatively limited, indicating that the rainfall-triggering mechanism of landslides in Hong Kong is primarily centered on short-duration intense rainfall processes, supplemented by the combined influence of extreme precipitation events.

**Figure 9:**
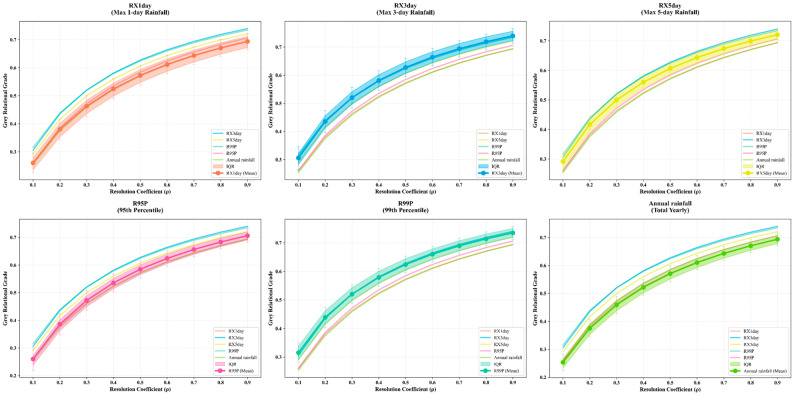
Sensitivity analysis of GRA relational grades for six rainfall indices under different distinguishing coefficients (τ = 0.1–0.9).

**Figure 10:**
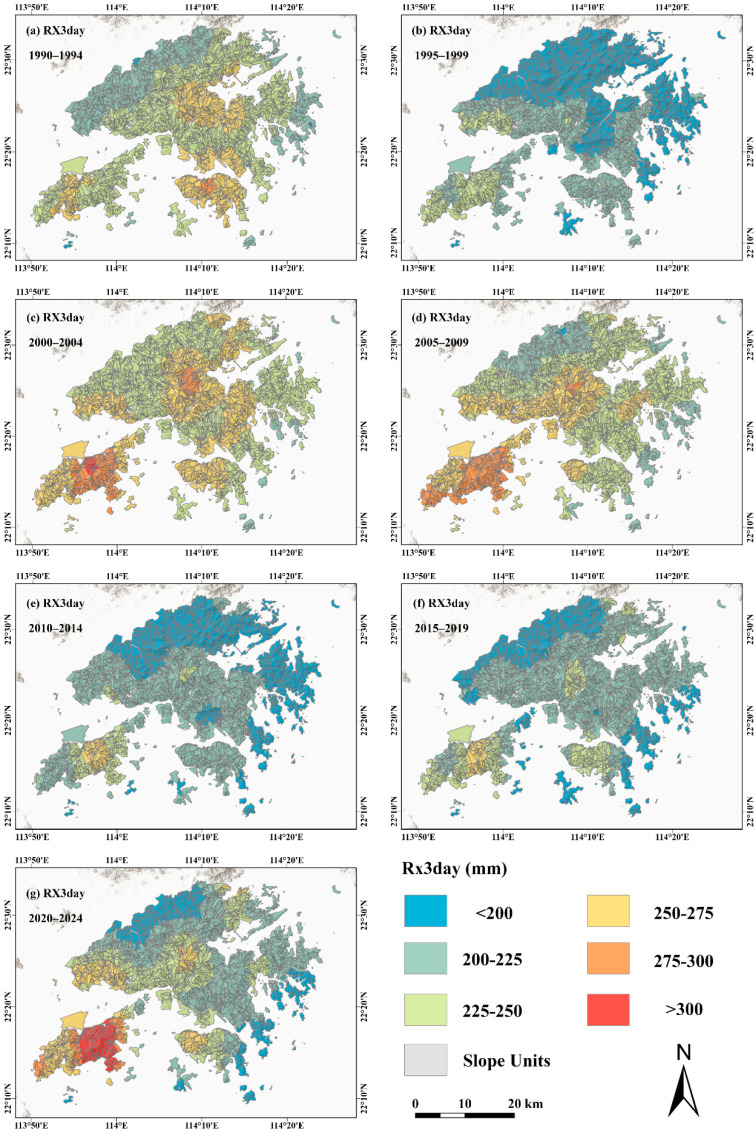
(**a**–**g**) Rx3day rainfall index aggregated at five-year intervals during 1990–2024.

In grey relational analysis, the value of the distinguishing coefficient τ affects the calculation of grey relational coefficients and may consequently influence the identification of the dominant rainfall index. To examine the robustness of the analytical results, a systematic sensitivity analysis was conducted for the 1923 slope units with landslide occurrences by varying τ from 0.1 to 0.9 with an interval of 0.1, and the grey relational degrees of the six rainfall indices were recalculated under each parameter setting. Figure 9 illustrates the variation in the mean grey relational degrees of different rainfall indices under varying distinguishing coefficients, where thick solid lines with filled circles represent mean values, shaded areas denote the interquartile range (IQR, 25–75%), and error bars indicate standard deviations. The results show that the mean relational degrees of all rainfall indices exhibit a monotonic increasing trend with increasing τ, with values ranging from 0.25–0.32 at τ = 0.1 to 0.69–0.74 at τ = 0.9. Although the absolute values of the relational degrees increase systematically with changes in τ, the relative ranking among the rainfall indices remains highly consistent across all parameter settings, indicating that variations in the distinguishing coefficient affect the absolute magnitude of the relational degrees but do not alter the relative strength of association among the rainfall indices. Among them, RX3day consistently exhibits the highest mean relational degree under all distinguishing coefficient values and remains ranked first throughout, showing a clear advantage over the other rainfall indices and demonstrating strong stability and insensitivity to parameter variation. The ranking of the remaining five rainfall indices also shows no structural changes. Overall, the systematic sensitivity analysis based on τ = 0.1–0.9 indicates that variations in the distinguishing coefficient do not change the identification of the dominant rainfall index, further confirming the robustness and reliability of the grey relational analysis results. Therefore, RX3day can be confirmed as the rainfall index that is most strongly and stably associated with landslide activity at the long-term scale in the study area (Figure 10).

### 4.3. Landslide Driving Analysis Based on the Optimal Parameter-Based Geographical Detector

**Figure 11:**
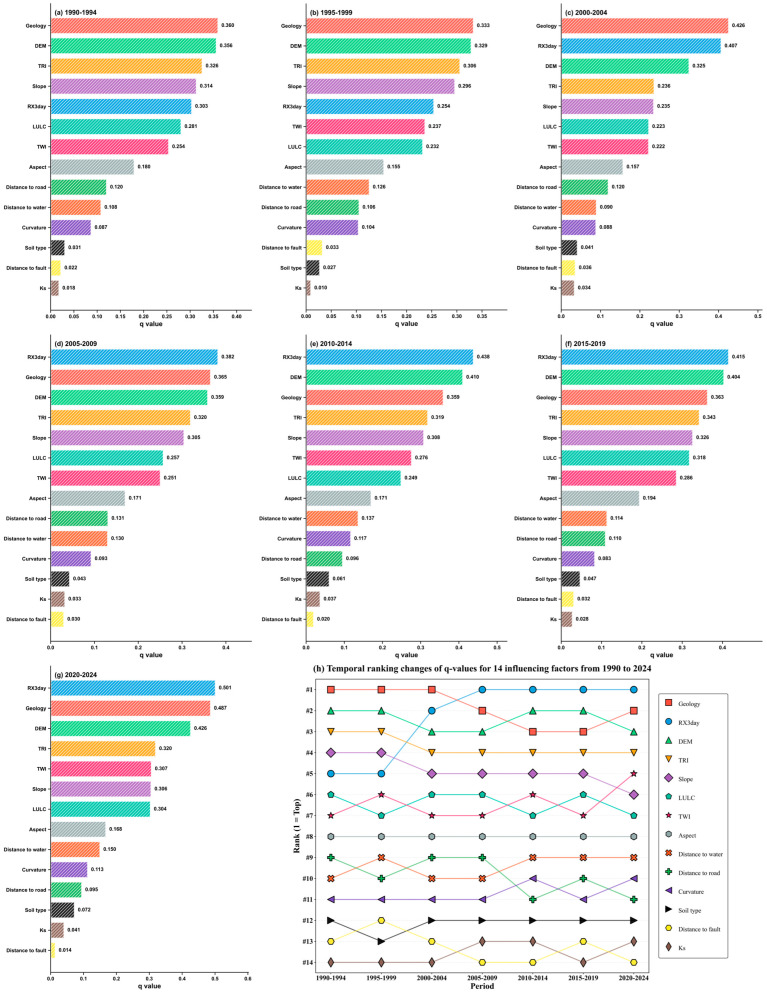
(**a**–**g**) Factor detection results at five-year intervals during 1990–2024; (**h**) temporal variation in factor rankings from 1990 to 2024.

After identifying RX3day as the dominant rainfall index, the optimal parameter-based geographical detector (OPGD) was further applied to quantitatively analyze the multi-factor driving characteristics of landslide spatial distribution across seven consecutive five-year periods from 1990 to 2024. Based on the single-factor detection results (Figure 11), the relative explanatory power of 14 landslide-related factors on landslide spatial heterogeneity was systematically evaluated. The results indicate that, over the entire 35-year study period, all influencing factors passed the significance test (p < 0.05), with q values generally ranging from 0.01 to 0.50, demonstrating that the spatial distribution of landslides in Hong Kong exhibits pronounced spatial heterogeneity and is jointly controlled by multiple environmental factors. From a long-term perspective, Geology, DEM, and RX3day consistently show relatively high explanatory power, forming a group of high-contribution driving factors for landslides. Among them, Geology and DEM remain stably ranked at the top during most periods, reflecting the long-term and stable fundamental constraints imposed by geological conditions and topographic framework on landslide spatial patterns; in contrast, the explanatory power of RX3day increases markedly during the middle and later periods. Slope and TRI maintain moderate-to-high q values across all periods, indicating that slope conditions and terrain roughness exert a persistent control on landslide occurrence. The explanatory power of LULC and TWI is generally at a moderate level but exhibits noticeable stage-wise fluctuations over time, suggesting clear temporal variability in their influence on landslide susceptibility. In comparison, factors such as Aspect, Distance to road, Distance to drainage, Curvature, Soil type, Distance to fault, and Ks show relatively low overall contributions, with q values remaining at low levels throughout the study period and only limited variations observed in individual periods, mainly reflecting secondary modulation effects on regional-scale landslide spatial heterogeneity. Under the static environmental background dominated by geological and topographic conditions, the dynamic triggering effect of extreme precipitation represented by RX3day shows a continuously strengthening trend. Its q value increases gradually during the early stage of the study period and enters a phase of pronounced enhancement around 2000; after 2005, RX3day progressively becomes one of the primary driving factors and reaches approximately 0.50 in 2020–2024, indicating that extreme rainfall has become an important triggering factor promoting landslide occurrence and significantly influencing their spatial distribution patterns. Combined with the factor ranking changes shown in Figure 11h, the landslide driving structure in the study area exhibits clear temporal evolution characteristics: Geology occupies a dominant position during the 1990–1999 period, whereas the ranking of RX3day increases significantly after 2000 and remains in the leading position after 2005; meanwhile, DEM, TRI, and Slope consistently remain at the forefront, jointly constituting the topographic conditions associated with landslide occurrence. In contrast, LULC and TWI mostly fall within the middle-ranking range and display stage-wise fluctuations; the explanatory power and ranking of Aspect remain relatively stable throughout the study period; and the low-contribution factors, including Distance to road, Distance to drainage, Curvature, Soil type, Distance to fault, and Ks, are generally ranked lower with limited temporal variation.

Overall, the explanatory power of different environmental factors on the spatial distribution of landslides in Hong Kong exhibits clear stage-wise evolution from 1990 to 2024, with geological and topographic factors consistently maintaining high contribution levels, while the explanatory power of extreme precipitation factors gradually strengthens over time.

**Figure 12:**
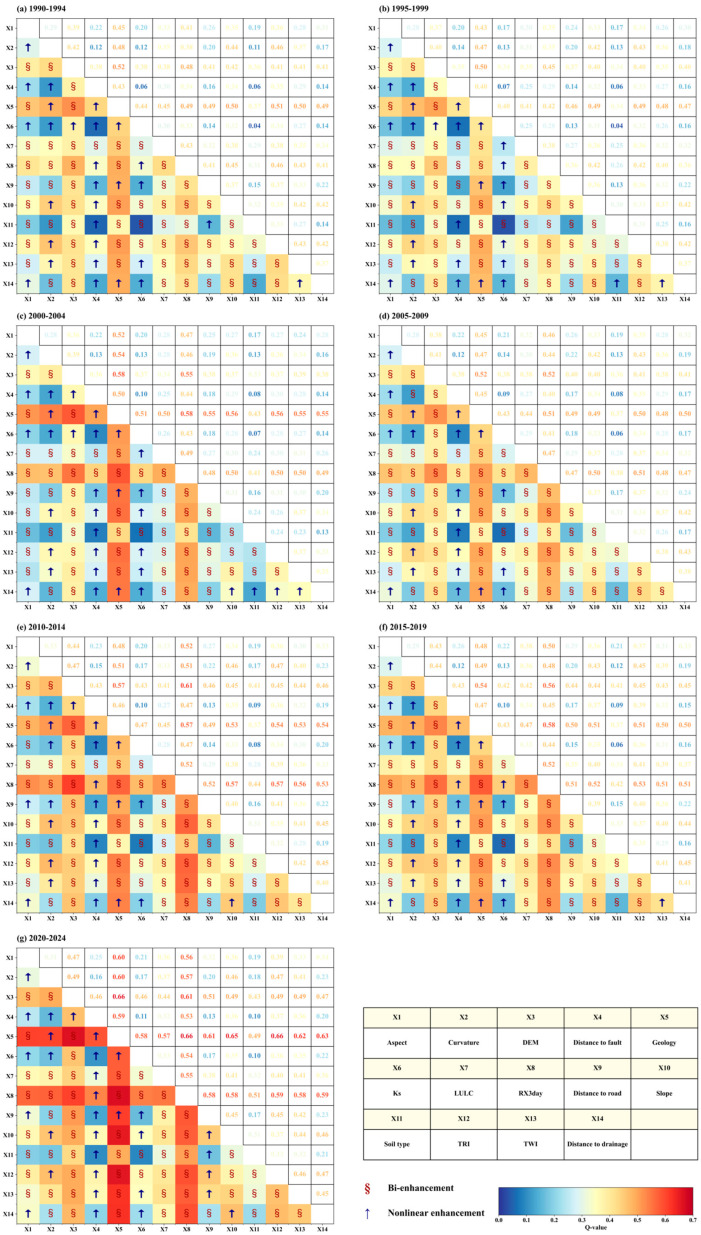
(**a**–**g**) Interaction detector results at five-year intervals during 1990–2024.

On the basis of clarifying the explanatory power of individual environmental factors on the spatial distribution of landslides, interaction detection was further conducted to analyze the synergistic effects of different factor combinations and to reveal the influence of multi-factor coupling on landslide spatial heterogeneity. Figure 12 presents the matrix distributions of pairwise factor interaction explanatory power and the corresponding interaction types for each five-year period from 1990 to 2024. Overall, all factor combinations passed the significance test (p < 0.05), and the interaction types were limited to “bivariate enhancement” and “nonlinear enhancement”, with no cases of weakening or independence, indicating that the spatial heterogeneity of landslides in Hong Kong is statistically characterized primarily by multi-factor coupling effects. From an overall structural perspective, high-value interaction zones in each period exhibit clear clustering patterns within the matrices. On the one hand, interactions between Geology and major topographic factors (e.g., DEM, TRI, Slope, and TWI) consistently remained within the high-value range throughout the study period, highlighting the fundamental role of geological conditions and topographic background in multi-factor coupling. On the other hand, since the 2000–2004 period, the interaction explanatory power between RX3day and Geology as well as other topographic factors has increased markedly, gradually forming more pronounced high-value clusters in subsequent stages. In terms of peak interaction strength, the maximum interaction q value increased from 0.52 in 1990–1994 to 0.66 in 2020–2024, indicating that over time not only did the extreme level of interaction explanatory power continue to rise, but the proportion of high-explanatory interaction combinations within the overall structure also increased. Period-by-period comparisons further reveal both the stability and stage-wise evolution of high-intensity interaction combinations. During 1990–1999, high-value interactions were mainly dominated by “geology–topography” combinations, with DEM∩Geology, TRI∩Geology, Slope∩Geology, and TWI∩Geology consistently ranking among the highest. A marked shift occurred during 2000–2004, when the interaction explanatory power of RX3day∩Geology rose to the highest level of that period and, together with DEM∩Geology, formed the leading interaction combinations, indicating that rainfall-related interactions began to enter the high-value range; this feature was further strengthened in subsequent stages. By 2020–2024, high-value interaction zones became increasingly concentrated within an interaction framework centered on RX3day, Geology, and major topographic factors, with RX3day∩Geology and DEM∩Geology stably occupying the top two positions, followed by TRI∩Geology and Slope∩Geology. This pattern indicates that, in the later stages of the study period, high-explanatory interactions governing the spatial distribution of landslides were primarily contributed by the coupled effects of extreme rainfall and geological–topographic conditions.

**Figure 13:**
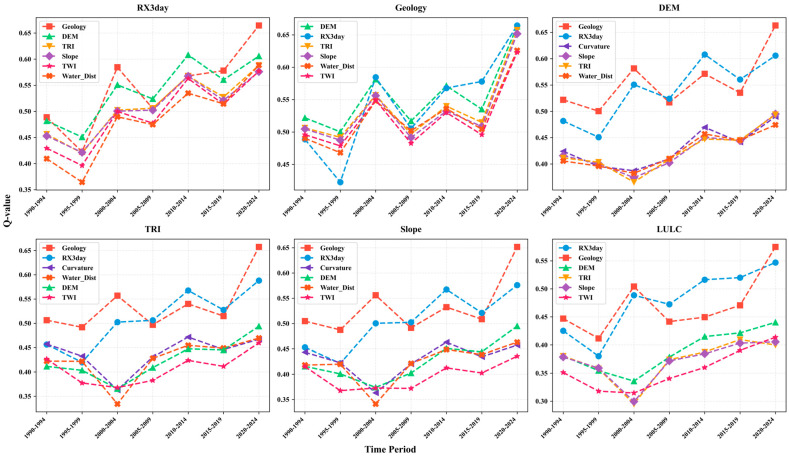
q-value rankings of the top six dominant factors and their top six factor interactions.

To further highlight the dominant role of core driving factors at the interaction level, Figure 13 summarizes and compares the temporal variations in interaction explanatory power (q values) between the core factors and their primary interaction counterparts across different five-year periods. The results show that the strongest interaction partner of RX3day consistently corresponds to Geology or DEM in all periods, with the maximum interaction q value increasing steadily from 0.49 in 1990–1994 to 0.66 in 2020–2024, indicating a pronounced strengthening over time of the coupled effects between extreme rainfall and geological–topographic conditions. For Geology, the strongest interaction partners in the early periods were mainly DEM and TRI; however, since 2000–2004, RX3day has gradually become its dominant interaction counterpart, reaching a q value of 0.66 in 2020–2024. This shift reflects the increasing modulation of landslide spatial patterns by rainfall factors under persistent geological constraints. Regarding topographic factors, such as TRI, Slope, and TWI, their strongest interaction partners in each period were predominantly Geology or RX3day, and the peak interaction q values for these combinations increased synchronously in the later stages of the study period. This pattern indicates a synergistic amplification effect of topographic conditions under an increasingly strengthened rainfall-triggering regime. In addition, some distance-related factors and LULC also entered relatively high explanatory-power tiers when coupled with core factors. For example, the q value of Geology∩Distance to drainage increased from 0.49 to 0.63, and that of Geology∩Distance to road increased from 0.49 to 0.61. Interactions between LULC and Geology or RX3day also strengthened during the middle and later periods, although their explanatory power remained lower than that of the highest-value combinations, such as RX3day∩Geology and DEM∩Geology.

By synthesizing the results shown in Figure 11 and Figure 12, it can be observed that high-explanatory-power interactions underlying landslide spatial heterogeneity during the study period exhibit a clear and stable dominant structure. Interactions between Geology and major topographic factors form a long-term foundational framework characterized by consistently high explanatory power, whereas interactions associated with RX3day have intensified markedly since around 2000 and reached their peak in recent periods. At the interaction level, these findings further corroborate and refine the conclusions derived from the single-factor detection, indicating that the spatiotemporal pattern of landslides in Hong Kong is jointly shaped by long-term stable geological–topographic background conditions and a markedly strengthened extreme rainfall triggering effect over the past decades. Meanwhile, some secondary factors contribute in a differentiated manner across periods, primarily through their coupling with the core driving factors.

## 5. Discussion

### 5.1. Dominant Rainfall Index and Multi-Factor Driving Mechanisms

From the perspective of spatiotemporal consistency, this study proposes an objective approach for identifying the rainfall index most strongly associated with landslide activity. In previous landslide studies in Hong Kong, the selection of rainfall factors has often relied on empirical judgment, single-index inputs, or specific model settings. Although such approaches may be reasonable for particular periods, they are prone to introducing index redundancy and multicollinearity in long-term analyses, thereby weakening the stability and comparability of interpretations of landslide driving mechanisms. By incorporating Grey Relational Analysis at the slope-unit scale, this study systematically compares the temporal and spatial response characteristics of multiple candidate rainfall indices, providing a data-driven and reproducible solution for rainfall index optimization. This process helps to standardize the representation of rainfall-triggering factors for landslides over long time scales, reduces uncertainties associated with the parallel use of different rainfall indices, and establishes a stable dynamic triggering variable for subsequent analyses of landslide driving mechanisms. Compared with approaches that assess the importance of rainfall variables only during the model construction stage, this method clarifies the representativeness of rainfall indices prior to driver analysis, thereby enhancing the physical consistency of the interpreted driving factors.

On this basis, the optimal parameter-based geographical detector was further employed to conduct a unified analysis of landslide driving factors across seven consecutive five-year periods from 1990 to 2024 at the slope-unit scale, enabling a systematic characterization of the long-term evolution of landslide driving mechanisms from a spatiotemporal perspective. Unlike traditional studies that rely on single time periods or post hoc model-based interpretations, this framework allows the explanatory power of individual environmental factors on landslide spatial distribution to be directly quantified for different time stages and compared across periods, thereby more clearly revealing the temporal evolution and structural changes of landslide driving mechanisms.

### 5.2. Comparison with Existing Studies

In terms of rainfall index selection, previous landslide studies in Hong Kong have paid relatively limited attention to the spatiotemporal association analysis of quantitative rainfall indices. Many studies have directly adopted annual total rainfall as a proxy factor [13,14], focused on short-term triggering analyses based on single extreme rainstorm events [10,28], or simultaneously incorporated multiple rainfall indices into models as parallel environmental variables without systematic screening [11,12]. Although these approaches may be applicable under specific circumstances, they are susceptible to multicollinearity among indices, spatiotemporal inconsistency, and subjective selection, which constrains the robust characterization of landslide triggering mechanisms at long-term scales. In this study, Grey Relational Analysis was employed to conduct an objective and systematic comparison of multiple candidate rainfall indices from both temporal and spatial perspectives, leading to the identification of RX3day as the dominant triggering index. This data-driven approach provides a new pathway for the quantification and spatial representation of rainfall characteristics in landslide studies, enabling the establishment of a consistent dynamic triggering variable at a macroscopic long-term scale. Moreover, this study is complementary to the application of Grey Relational Analysis by Yan et al. [20]: while the latter primarily focused on the relationship between rainfall indices and landslide density, the present study further extends the analysis to slope-unit-scale spatial heterogeneity and 35-year evolutionary characteristics, achieving a more comprehensive assessment of spatiotemporal consistency.

In terms of driving mechanisms, the results of this study are consistent with previous research in showing that landslides in Hong Kong are jointly controlled by geological–topographic conditions as a stable background and extreme rainfall as a dynamic trigger. However, most earlier studies were based on single rainstorm events, short-term datasets, or static models, which limited their ability to capture the temporal evolution of driving factors [7,8,9,14,15]. By applying the Optimal Parameter-based Geographical Detector within a unified, long-term, stage-based analytical framework, this study reveals a pronounced increase in the explanatory power of extreme rainfall, as well as a continuous strengthening of the interaction effects between rainfall and geological/topographic conditions. This spatiotemporal characterization is more systematic and comprehensive than previous studies focusing on specific events or short-term periods, and it reveals the progressively intensifying influence of extreme rainfall in Hong Kong over a multi-year timescale.

### 5.3. Limitations and Future Research Directions

Although this study establishes an integrated framework at the slope-unit scale for selecting the rainfall index most strongly associated with landslide activity and for analyzing multi-factor spatiotemporal driving mechanisms, and systematically reveals the dominant rainfall factor and multi-factor controls of landslide activity in Hong Kong during 1990–2024, several limitations remain that warrant further investigation.

First, with respect to rainfall factor selection, this study mainly focuses on six representative rainfall indices commonly used in landslide research (e.g., RX1day, RX3day, RX5day, and extreme precipitation percentile indices) to ensure comparability and a solid research basis among different indices. However, rainfall processes exhibit pronounced multi-scale and multi-characteristic attributes, and other potential quantitative representations of rainfall characteristics were not included in the analysis. Second, regarding the characterization of landslide driving mechanisms, this study primarily elucidates the stage-wise temporal evolution of the landslide driving structure in Hong Kong from 1990 to 2024, clarifying the relative changes between the static geological–topographic background and the dynamic triggering effect of extreme rainfall. Nevertheless, the results mainly emphasize the overall temporal evolution of driving mechanisms and do not further delineate, from a spatial perspective, the distribution of high-susceptibility areas corresponding to different driving structures.

Overall, this study provides a reproducible integrated framework for long-term spatiotemporal analysis of landslide driving mechanisms in Hong Kong. Future work that expands the rainfall index system and strengthens the spatial expression of driving-mechanism results is expected to further deepen understanding of landslide risk assessment in Hong Kong and to provide more targeted scientific support for landslide prevention, mitigation, and disaster-risk management.

## 6. Conclusions

This study adopts slope units as the fundamental analytical units to systematically identify and validate the dominant rainfall index and multi-factor driving mechanisms of landslide activity in Hong Kong. Grey relational analysis based on the 1990–2024 landslide inventory and multiple rainfall indices indicates that RX3day consistently exhibits the strongest and most stable association with landslide activity under different distinguishing-coefficient settings, with its ranking showing low sensitivity to parameter variation. This demonstrates that RX3day effectively captures the key rainfall characteristics governing landslide triggering processes in Hong Kong across both spatial and temporal scales, and can be regarded as the most representative quantitative rainfall index for the study area. On this basis, the optimal parameter-based geographical detector was employed to systematically analyze the long-term spatiotemporal evolution of landslide driving factors. The results show that geological and topographic factors (e.g., lithology, DEM, and terrain ruggedness index) constitute stable background controls on landslide distribution, whereas the explanatory power of the extreme rainfall factor RX3day has increased markedly since around 2000 and has evolved into one of the dominant dynamic driving factors in recent periods. This pattern reflects a sustained strengthening of rainfall-triggering effects at the multi-decadal scale. Interaction detection further reveals that the spatial heterogeneity of landslides in Hong Kong is primarily governed by nonlinear multi-factor coupling effects. Specifically, “geology–topography” combinations establish the fundamental spatial pattern of landslide susceptibility, while the progressively strengthening “rainfall–geology/topography” interactions represent a key mechanism underlying the recent increase in landslide risk. These findings indicate that landslide activity is not controlled by a single factor, but rather by the combined effects of long-term background conditions and dynamic triggering processes.

Despite establishing an integrated framework for rainfall-index selection and long-term multi-factor driving-mechanism analysis, and systematically revealing the dominant controls on landslide activity in Hong Kong over the past three decades, several limitations remain. The rainfall index system is still mainly composed of commonly used representative indices and does not fully capture the multi-scale characteristics of rainfall processes. In addition, the driving-mechanism analysis emphasizes temporal evolution, while the spatial differentiation of landslide conditioning factors under different driving structures requires further investigation. Future studies may expand the rainfall-index system and, from a spatial perspective, further elucidate how different landslide conditioning factors (e.g., geological background, topographic conditions, and extreme rainfall) influence landslide occurrence and shape the spatial differentiation of dominant driving structures, thereby deepening understanding of landslide hazard mechanisms in Hong Kong and supporting more refined risk management.

## Figures and Tables

**Table 1:** Information on Landslide conditioning factors.

Data Type	Format	Spatial Resolution	Source
DEM	Raster	5 m × 5 m	https://www.landsd.gov.hk/en/spatial-data/open-data.html, accessed on 24 February 2026
Slope	Raster	5 m × 5 m	DEM inversion
Aspect	Raster	5 m × 5 m	DEM inversion
Curvature	Raster	5 m × 5 m	DEM inversion
Geology	Raster	30 m × 30 m	https://www.cedd.gov.hk/filemanager/eng/share/map/geo_map_2.html, accessed on 24 February 2026
Soil type	Raster	1000 m × 1000 m	https://www.fao.org/soils-portal/data-hub/soil-maps-and-databases/harmonized-world-soil-database-v20/en/, accessed on 24 February 2026
TWI	Raster	5 m × 5 m	DEM inversion
TRI	Raster	5 m × 5 m	DEM inversion
Distance to road	Raster	5 m × 5 m	https://data.gov.hk/en-data/dataset/hk-td-tis_15-road-network-v2, accessed on 24 February 2026
Distance to drainage	Raster	5 m × 5 m	https://portal.csdi.gov.hk/geoportal/#metadataInfoPanel, accessed on 24 February 2026
Distance to fault	Raster	5 m × 5 m	https://www.cedd.gov.hk/filemanager/eng/share/map/geo_map_2.html, accessed on 24 February 2026
Soil saturated hydraulic	Raster	250 m × 250 m	https://www.futurewater.eu/projects/hihydrosoil-v2-0-global-maps-of-soil-hydraulic-properties-at-250m-resolution/, accessed on 24 February 2026
LULC	Raster	30 m × 30 m	https://zenodo.org/records/15853565, accessed on 24 February 2026
Rainfall	Raster	5500 m × 5500 m	https://www.chc.ucsb.edu/data/chirps3, accessed on 24 February 2026

**Table 2:** Cross-tabulation of slope unit counts based on landslide hotspot–cold spot and Mann–Kendall trend analyses.

Spatial Pattern	Up Trend	Down Trend	Not Significant	Total
Hot spot	13	158	810	981
Cold spot	0	1	358	359
Not significant	15	123	2953	3091
Total	28	282	4121	4431

## Data Availability

Data will be made available on request.
